# Supplementary material for: Carrier-Free Hybrid Nanoparticles for Enhanced Photodynamic Therapy in Oral Carcinoma via Reversal of Hypoxia and Oxidative Resistance
Source: Pharmaceutics. 2024 Aug 27;16(9):1130. doi: 10.3390/pharmaceutics16091130 (PMC11434982; doi:10.3390/pharmaceutics16091130)
Supplement: Supplementary file 1 [file pharmaceutics-16-01130-s001.zip › pharmaceutics-3076770-supplementary.pdf]

Supporting Information

Carrier-Free Hybrid Nanoparticles for Enhanced Photodynamic Therapy in Oral Carcinoma via Reversal of Hypoxia and Oxidative Resistance

Xiao Li [1,†], Zhiyin Li [2,†], Yue Su [3], Jia Zhou [4], Yuxiang Li [3], Qianqian Zhao [4], Xia Yang [2], Leilei Shi [5,*] and Lingyue Shen [2,*]

[1] Department of Cleft Palate Speech, Department of Oral and Maxillofacial Surgery, The First Affiliated Hospital of Harbin Medical University, Harbin 150001, China; lixiao@hrbmu.edu.cn

[2] Department of Oral and Maxillofacial-Head and Neck Oncology, Department of Laser and Aesthetic Medicine, Shanghai Ninth People's Hospital, Shanghai Jiao Tong University School of Medicine, College of Stomatology, Shanghai Jiao Tong University, National Center for Stomatology, National Clinical Research Center for Oral Diseases, Shanghai Key Laboratory of Stomatology, Shanghai Research Institute of Stomatology, Shanghai Center of Head and Neck Oncology Clinical and Translational Science, China; lizhiyin-1116-sjtu@sjtu.edu.cn (Z.L.); yangx2257@sh9hospital.org.cn (X.Y.)

[3] School of Chemistry and Chemical Engineering, Frontiers Science Center for Transformative Molecules, Shanghai Jiao Tong University, 800 Dongchuan Road, Shanghai 200240, China; yuesu@sjtu.edu.cn (Y.S.); lyx13062000501@sjtu.edu.cn (Y.L.)

[4] Department of Radiology, Shanghai Sixth People's Hospital Affiliated to Shang Hai Jiao Tong University School of Medicine, China; jia0531@sjtu.edu.cn (J.Z.); zhaoqianq5@sjtu.edu.cn (Q.Z.)

[5] Precision Research Center for Refractory Diseases in Shanghai General Hospital, Shanghai Jiao Tong University School of Medicine, Shanghai 200025, China.

* Correspondence: leilei.shi@shgh.cn (L.S.); shenly1703@sh9hospital.org.cn (L.S.)

† These authors have contributed equally to this work.

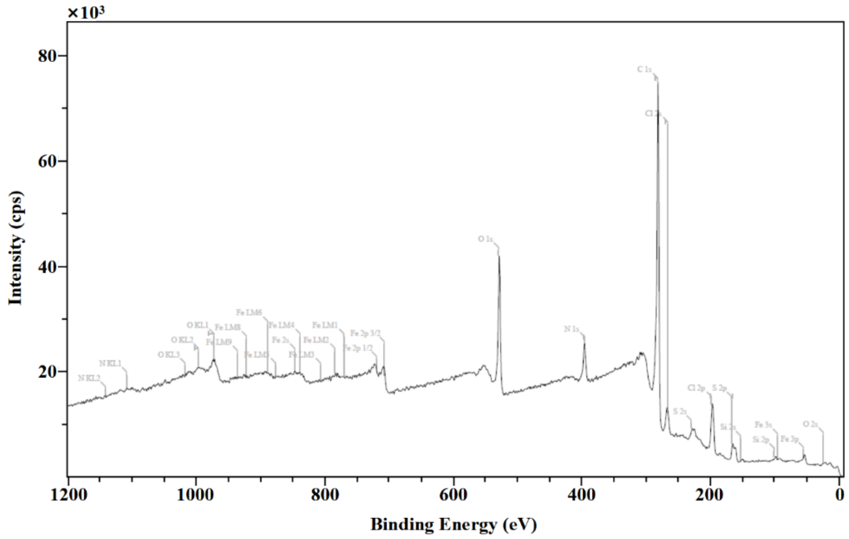

Figure S1. The XPS survey spectra of KPF NPs.

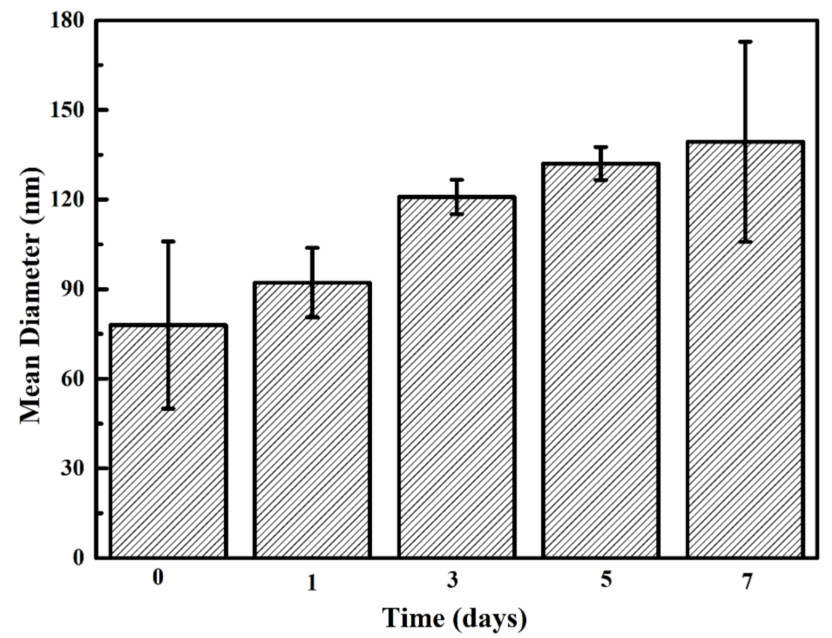

Figure S2. Size of the KPF NPs at different time intervals in PBS (pH = 7.4) determined by DLS. Error bars represent the standard deviation (n = 3).

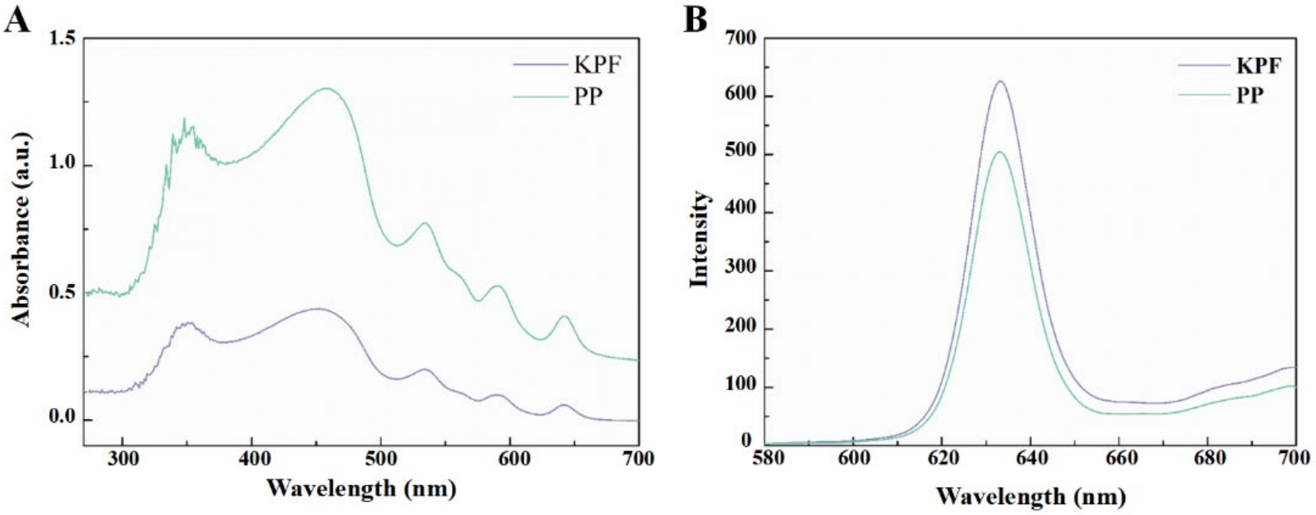

Figure S3. (A) UV–vis absorption spectra of KPF NPs and PP in H_2O. (B) KPF NPs and PP fluorescence spectra in PBS solvent.

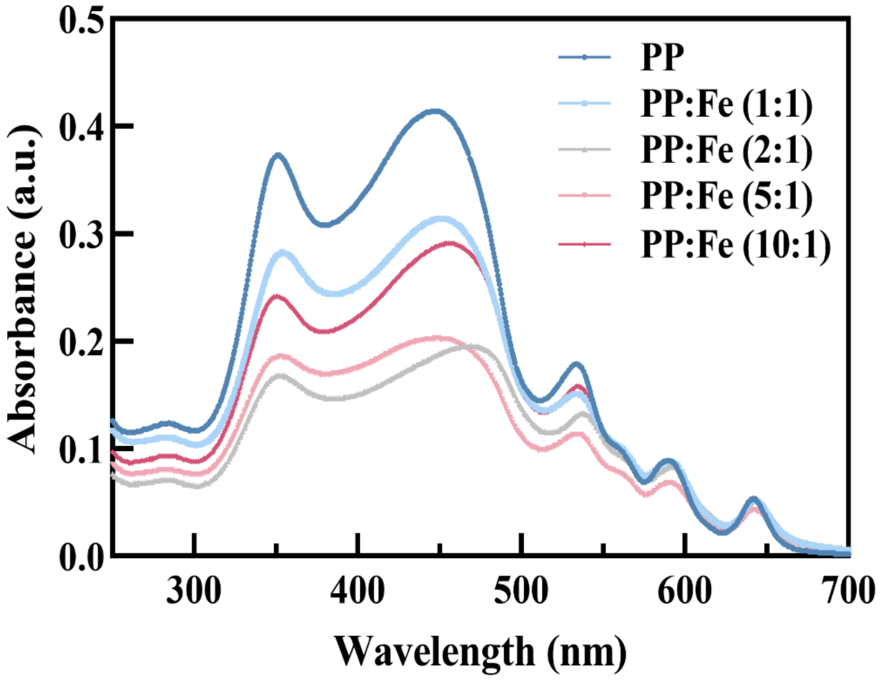

Figure S4. UV–vis absorption spectra of PP and Fe in different ratios.

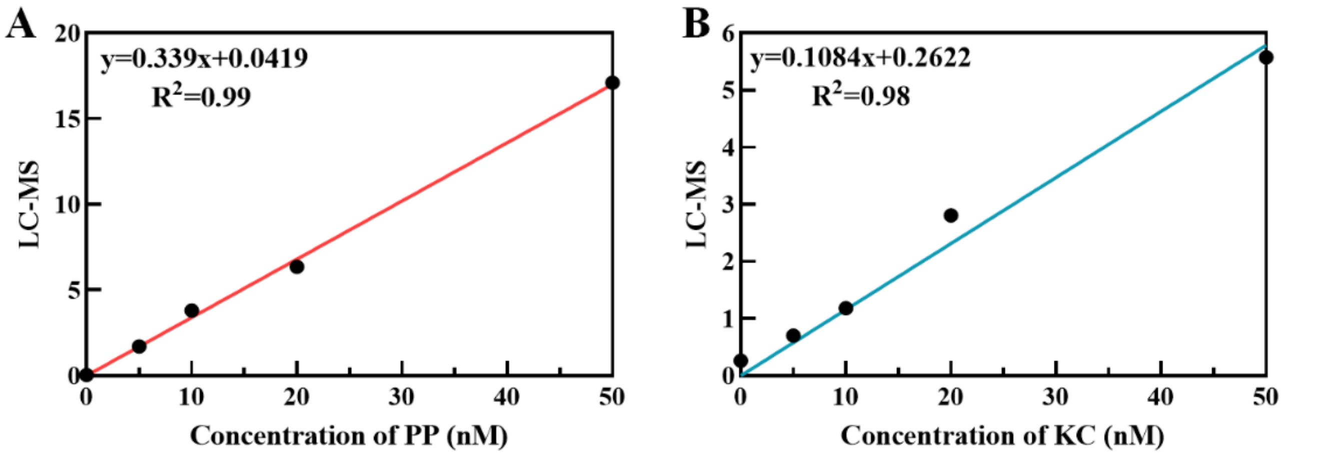

Figure S5. (A) The standard curve of PP in the KPF NPs was tested from LC-MS spectra. (B) The standard curve of KC in the KPF NPs was tested from LC-MS spectra.

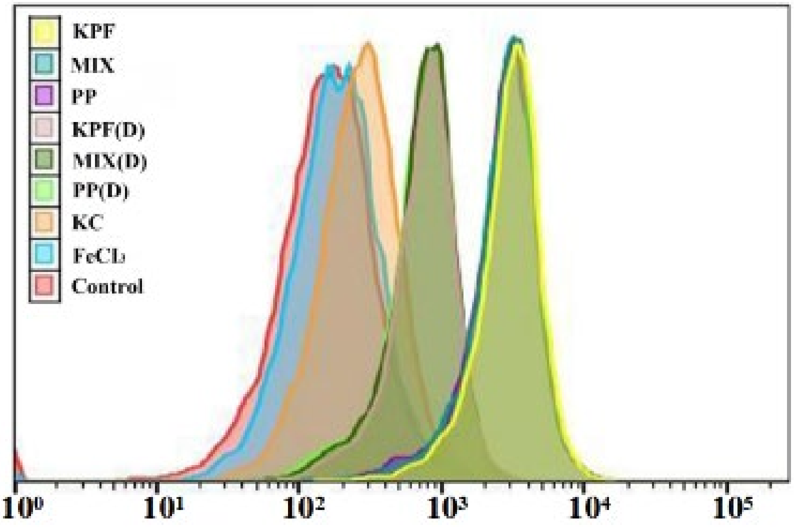

Figure S6. Flow cytometry analysis of ROS levels characterized by DCFH-DA probe in CAL-27 cells after co-culture under indicated treatments with or without laser irradiation.

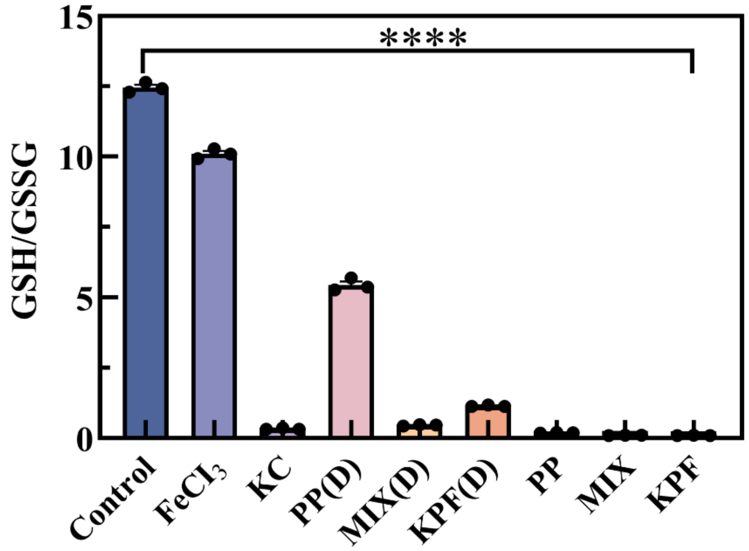

Figure S7. GSH/GSSG levels characterized in CAL-27 cells after co-culture under indicated treatments with or without laser irradiation.

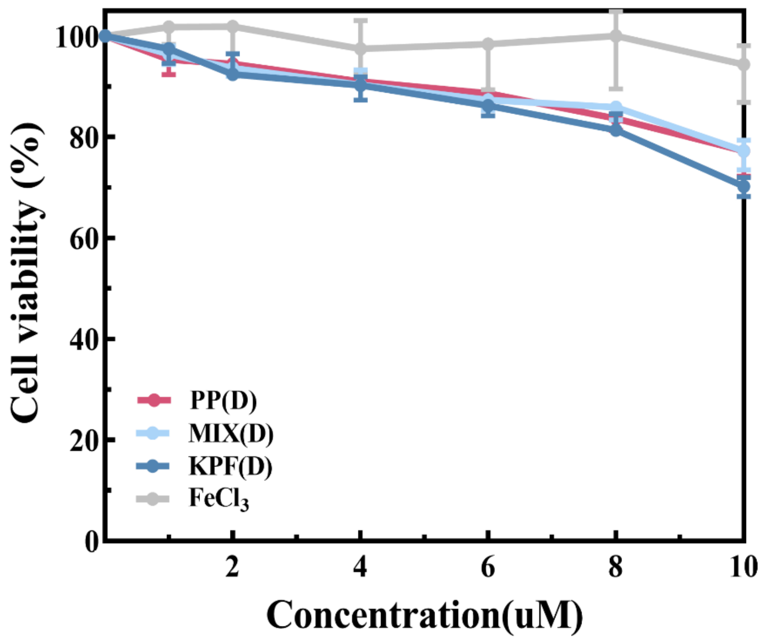

Figure S8. MTT assay of CAL-27 cells incubated with indicated solutions without irradiation for 24 h. The PP concentrations are 2μM, 4μM, 6μM, 8μM and 10μM, respec-tively. The KC7F2 concentrations are 1μM, 2μM, 3μM, 4μM and 5μM, respectively.

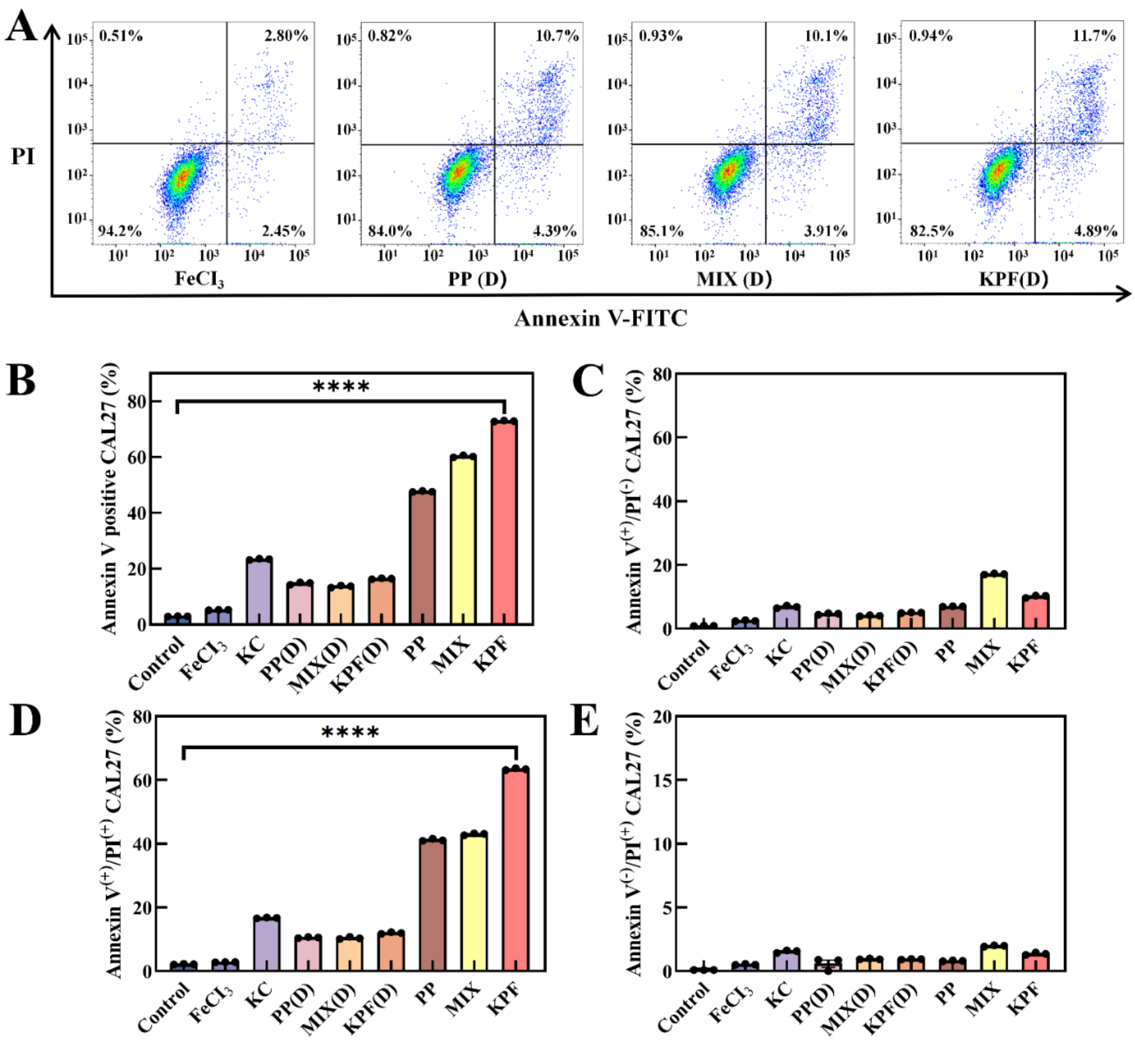

Figure S9. (A) Representative results from flow cytometric analysis of apoptosis in CAL-27 cells treated under indicated treatments without laser irradiation at equivalent concentrations of PP (8 μM) and KC7F2 (4 μM) for 24 h. (B) Ratio of apoptotic based on the results of flow cytometry measurements. (C) Ratio of early apoptosis based on the results of flow cytometry measurements. (D) Ratio of late apoptosis based on the results of flow cytometry measurements. (E) Ratio of necrosis based on the results of flow cytometry measurements. The results are expressed as mean ± SD of triplicate samples. "****" indicates means that are significantly different when compared to the control group ($P < 0.001$).

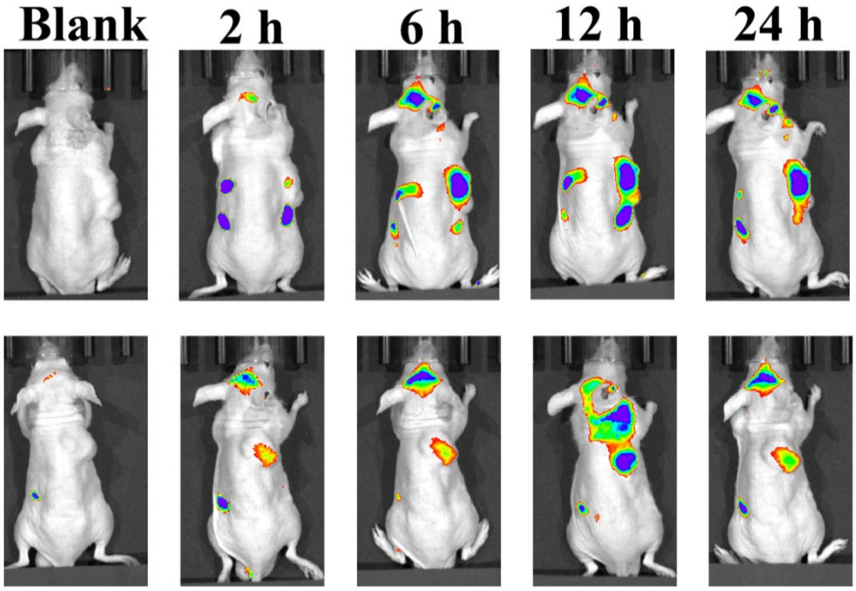

Figure S10. Real-time imaging of other two replicateons after the administration of KPF NPs (200 μL) at a dosage of 5 mg/kg corresponding to body weight.

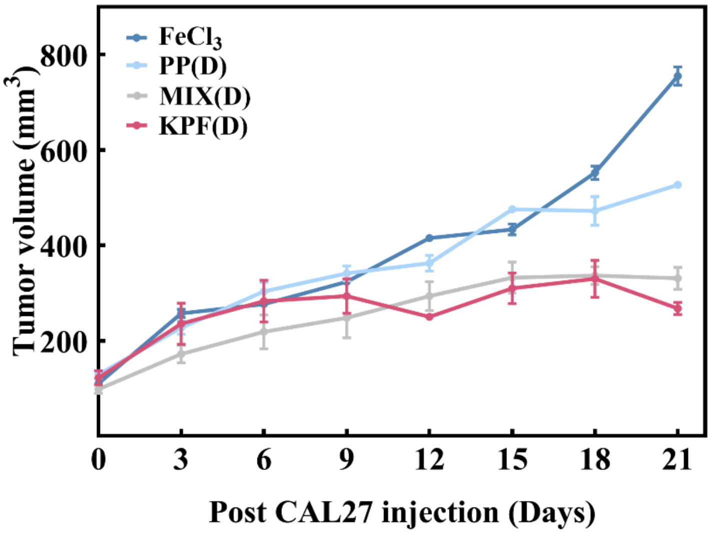

Figure S11. Tumor volume change after administration of KPF NPs (5 mg/kg based on PP, 200 μL), PP (5 mg/kg, 200 μL), PP/KC mixture, FeCl3 without irradiation.

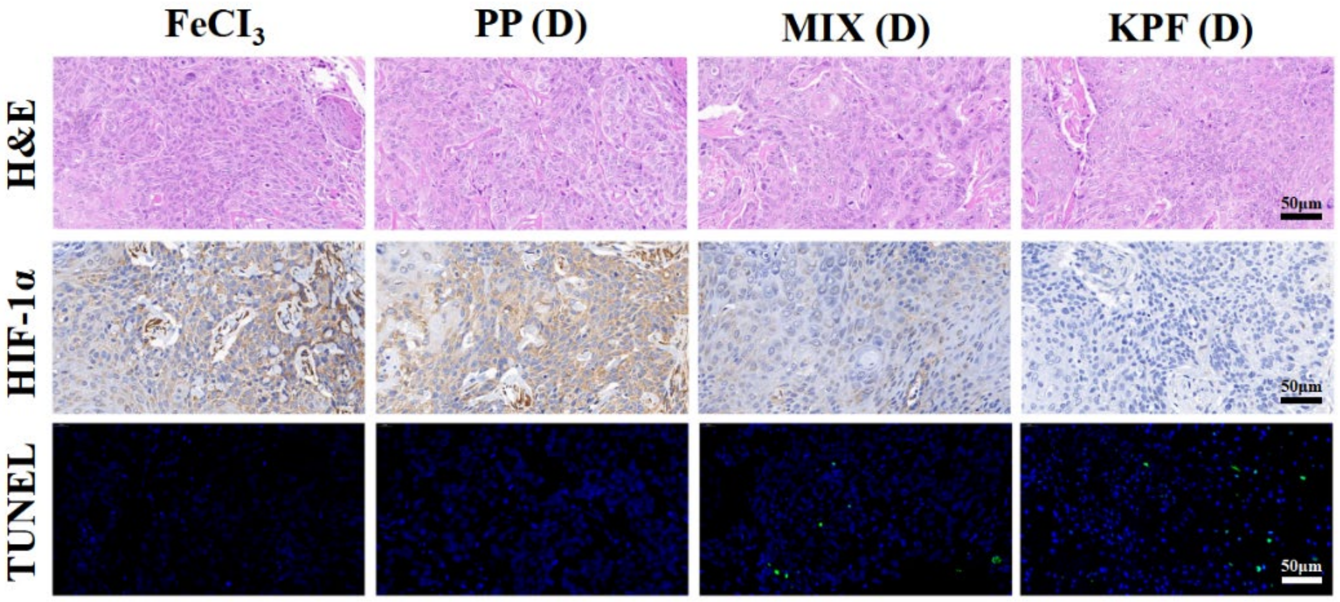

Figure S12. H&E analysis, immunohistochemistry analysis and histological immunofluorescence of tumor sections. Immunohistochemistry (IHC) analysis of HIF-1α expression in tumor tissues. Histological immunofluorescence in tumor sites labeled by TUNEL. Green: apoptosis cells. Blue: DAPI-stained cell nuclei. Scale bar is 50 μm.

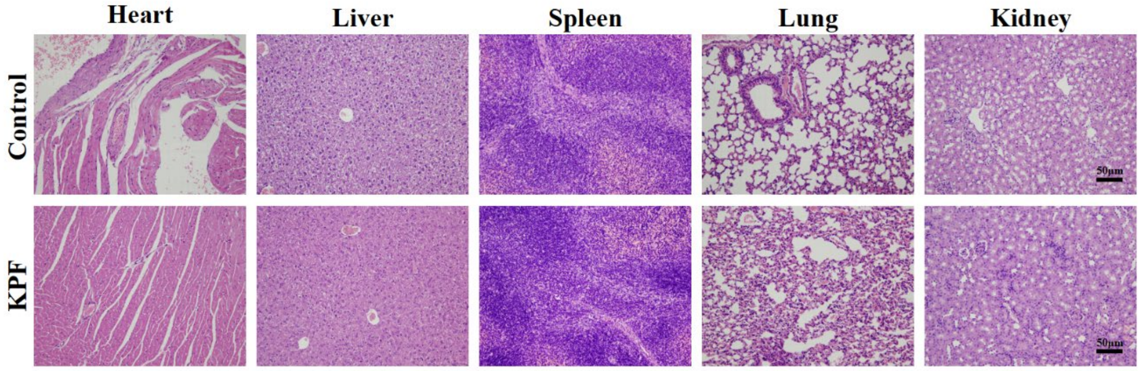

Figure S13. H&E analysis of different organs after mice were treated with PBS and KPF NPs (5 mg/kg).
